# Computational Study on the Dynamics of a Bis(benzoxazole)-Based Overcrowded Alkene

**DOI:** 10.1021/acs.jpca.4c06773

**Published:** 2025-01-23

**Authors:** Charlotte N. Stindt, Taegeun Jo, Jorn D. Steen, Ben L. Feringa, Stefano Crespi

**Affiliations:** † Stratingh Institute for Chemistry, University of Groningen, Nijenborgh 4, Groningen 9747 AG, the Netherlands; ‡ Department of Chemistry - Ångström Laboratory, Uppsala University, Box 523, Uppsala 751 20, Sweden

## Abstract

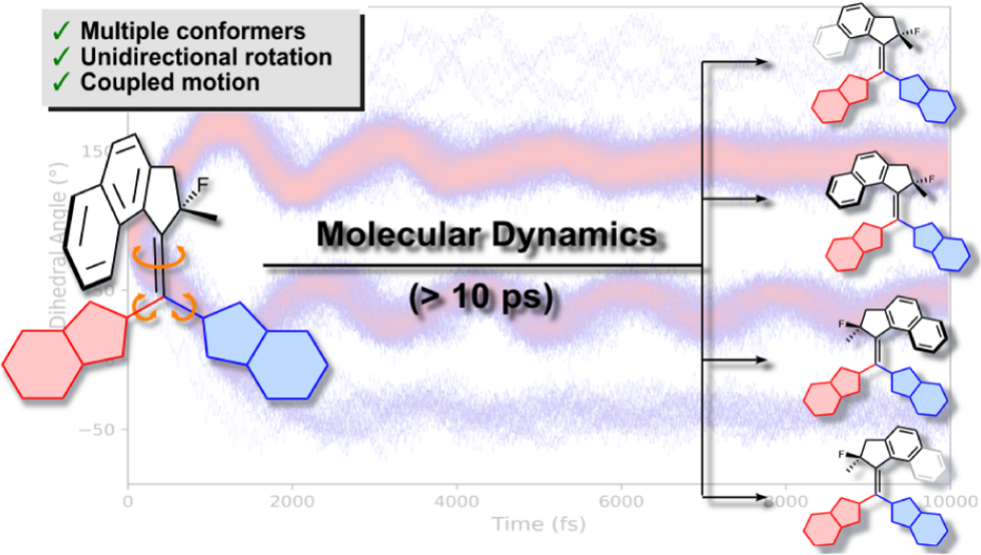

Understanding and controlling molecular motions is of pivotal importance for designing molecular machinery and functional molecular systems, capable of performing complex tasks. Herein, we report a comprehensive theoretical study to elucidate the dynamic behavior of a bis(benzoxazole)-based overcrowded alkene displaying several coupled and uncoupled molecular motions. The benzoxazole moieties give rise to 4 different stable conformers that interconvert through single-bond rotations. By performing excited- and ground-state molecular dynamics simulations, DFT calculations, and NMR studies, we found that the photochemical *E-Z* isomerization of the central double bond of each stable conformer is directional and leads to a mixture of metastable isomers. This transformation is analogous to the classical Feringa-type molecular motors, with the notable difference that, during the photochemical isomerization and the subsequent thermal helix inversion (THI) steps, multiple possible pathways take place that involve single-bond rotations that can be both coupled and uncoupled to the rotation of the naphthyl half of the molecule.

## Introduction

This year marks the 25th anniversary of the discovery of the first light-driven monodirectional molecular rotor.^1^ Since then, besides a large family of overcrowded alkene based rotary motors, a variety of other molecular motors has been designed.^2^ A few notable ones are the imine-based four-stroke and two-stroke motors developed by Lehn and coworkers for which the directionality is governed by the photochemical step;^3,4^ the hemithioindigo-based molecular motor first presented by Dube and coworkers in 2015,^5^ followed by several variations, including the first reported photon-only motor^6^ and a design that follows a figure-of-eight motion;^7^ the biomimetic^8^ and two-stroke^9^ motors from Olivucci and coworkers; and the recent molecular motors based on oxindole^10,11^ and barbituric acid^12^ by Feringa and Crespi.

**Figure 1:**
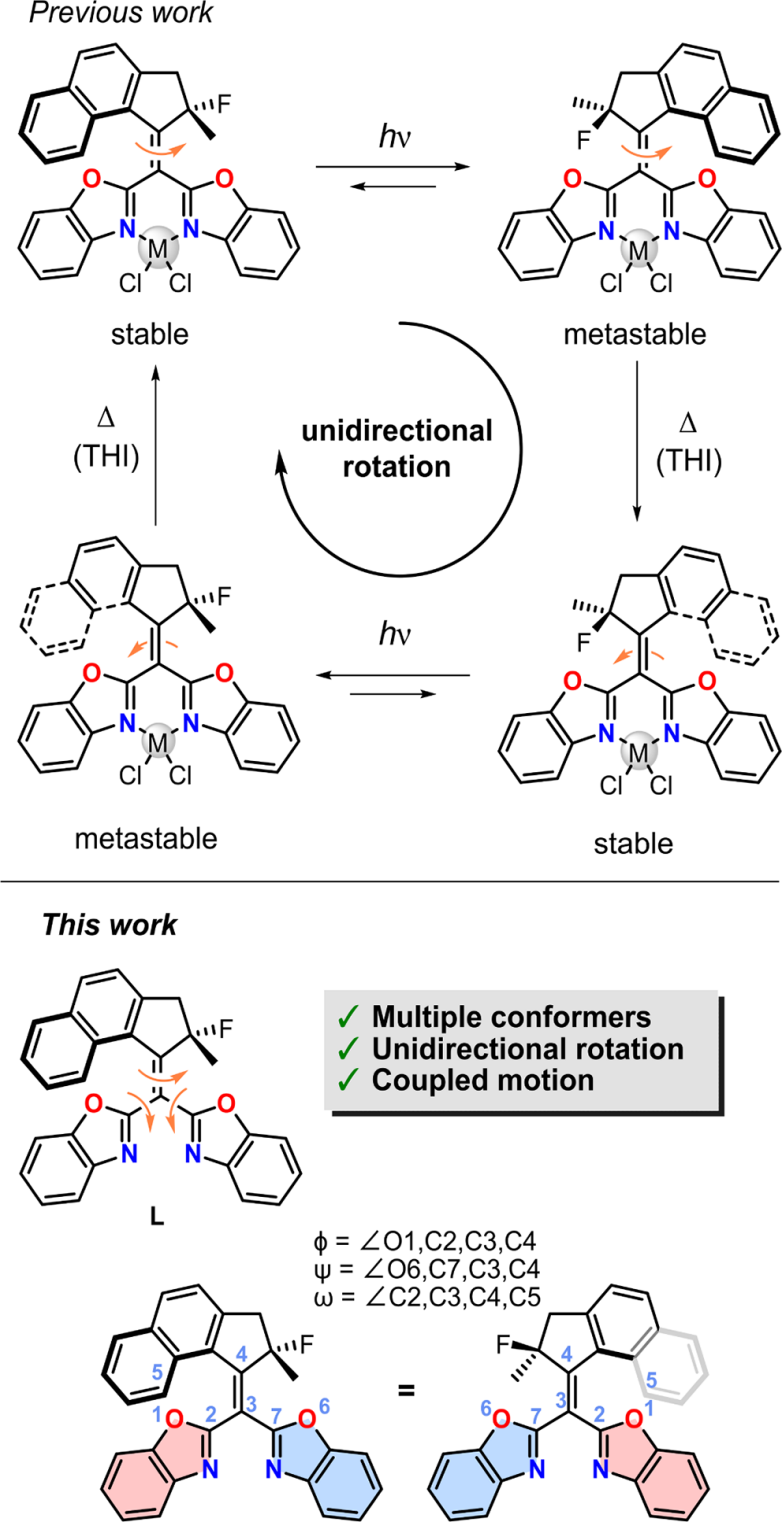
Motor ligand **L** can coordinate various metal salts which locks the rotating benzoxazole moieties in a single conformation and allows for the typical well-defined unidirectional rotation cycle consisting of two photochemical *E*-*Z* isomerizations and two thermal helix inversion (THI) steps. The three key dihedral angles (ψ, φ and ω) which are used for the assignment of conformers are highlighted.

Molecular motors of the Feringa type are based on overcrowded alkenes and consist of a rotator connected to a stator with a central C=C double bond (illustrated by the metallo-motor shown in Figure 1).^13^ In its rotary cycle, the stable isomers are converted to their respective metastable forms by a photochemical *E-Z* isomerization step which inverts the helicity of the motor and changes the orientation of the methyl group at the stereogenic center in the upper half from a pseudoaxial orientation to a sterically unfavored pseudoequatorial one (see Figure 1). The exact configuration of this stereocenter, together with the helical chirality, determines the direction of the rotation. The photochemical step is followed by a thermal helix inversion (THI) in which the rotator moves over the stator to relax to the next stable conformation in the cycle. In this step, the helicity is inverted again and the methyl group reassumes a pseudoaxial orientation. Subsequent photochemical and thermal steps complete the 360° rotation.

Since the discovery of light-driven rotary molecular motors based on overcrowded alkenes,^1^ the collection of motor structures has been greatly expanded and adapted for different applications.^14−19^ A key requisite for this achievement is the tremendous progress that has been made in the further development and fine-tuning of the motor properties, such as shifting the absorption wavelength toward the red,^20−22^ tuning the rotary speed,^23^ coupling various motions to the rotation of the upper half,^24−29^ and achieving *in situ* control over the motor properties.^30−33^ Recently, we reported a photochemically driven molecular motor based on a bis(benzoxazole) ligand **L**, that can coordinate various metal salts to achieve a well-defined motor function (Figure 1).^27^ Furthermore, by choosing different metal salts, the rotation speed and absorption wavelength can be tuned allowing for an unprecedented control of the molecular system. In contrast, without a “locking” agent, free rotation about the single bonds that attach the lower half to the central C=C bond (the axle of rotation) leads to the rapid interconversion of various conformers, which prohibits such a well-defined motor function.

Nevertheless, the complex behavior and dynamics of the noncoordinated **L** are intriguing. Due to the various conformations of the heterocyclic half, it represents a compound with potentially multiple photochemically and thermally induced molecular motions in a sterically very crowded environment. We assigned three key dihedral angles to characterize the movement of **L** (ψ, φ, and ω, the first two related to the motion of single bonds of the lower half and the third connected to the double bond rotation, see Figure 1).

Understanding and controlling the interplay between such molecular motions is a prerequisite for designing molecular machines and functional molecular systems for increasingly complex tasks. Here, we present a comprehensive computational study to elucidate the complex excited-state and ground-state thermal behavior of the bis(benzoxazole)-based overcrowded alkene **L**. We anticipate that shedding light on the dynamic behavior of molecules such as **L** will pave the way for the rational design of more intricate molecular systems and machinery.

## Results and Discussion

### Static Depiction of the Ground State

**Figure 2:**
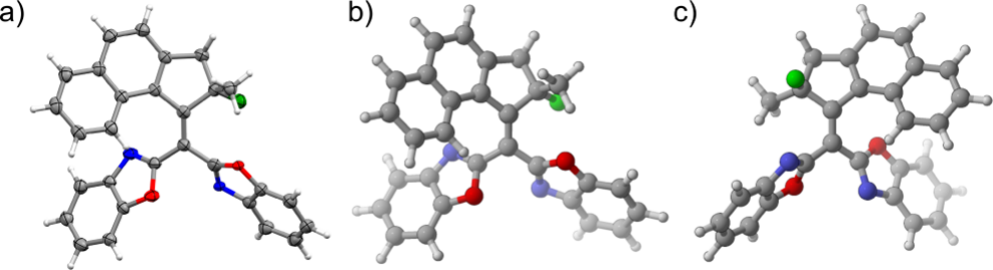
a) Crystal structure of **L** in its **L-S3** conformer. Ellipsoids are drawn at 50% probability. This figure has been previously reported in the literature.^27^ The crystallographic data have been deposited at the Cambridge Crystallographic Data Centre (CCDC) with deposition number [CCDC 2221929]. b) Optimized geometry of **L-S3** at the r^2^SCAN-3c level of theory. c) Optimized geometry of **L-M3** at the r^2^SCAN-3c level of theory.

First, ground-state geometry optimizations were performed on the various conformers using the composite method r^2^-SCAN-3c.^34^ It was found that **L** can adopt two different isomers around the overcrowded alkene double bond, which are analogous to the stable and metastable forms found in typical molecular motors based on overcrowded alkenes.^2^Figure 2 shows examples of these two forms for the calculated stable state (dubbed **L-S**, *vide infra* for a description of all minima found), featuring a pseudoaxial methyl group, as well as the metastable state **L-M**, with opposite helicity and a pseudoequatorial methyl group. The calculated structure matches very well with the structure that was previously found by single-crystal X-ray crystallography.^27^ Note that the single crystal X-ray structure in Figure 2a depicts only one stable conformation (**L-S3**) for clarity, whereas various stable conformers were present in the crystal.^27^ The crystal structure clearly showed the close resemblance of the ligand to a classical Feringa type-molecular motor in its stable form, including the helical structure of the ligand and the pseudoaxial orientation of the methyl group, suggesting the potential of the ligand to act as a unidirectional molecular motor.

**Figure 3:**
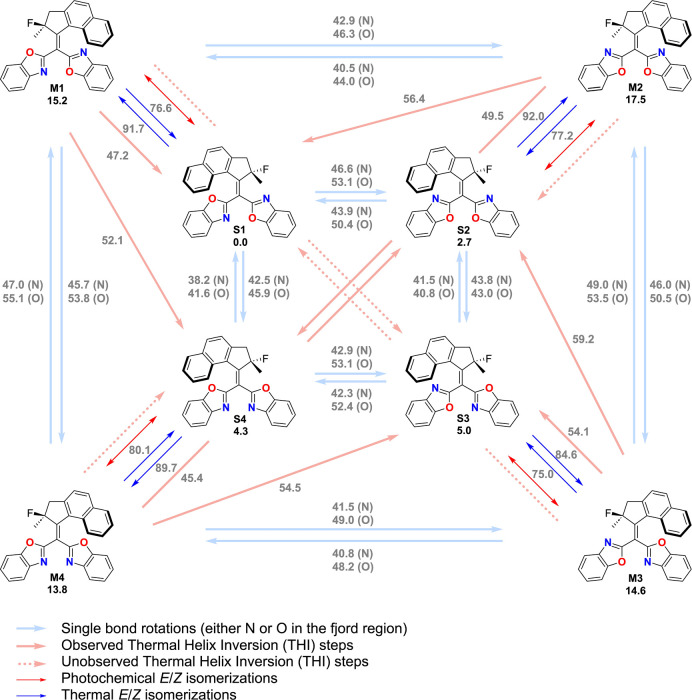
Overview of the possible interconversion pathways between the various stable (S1 to S4) and metastable (**M1** to **M4**) conformation of compound **L**, through various *E*-*Z* isomerizations, single-bond rotations and THI steps at the r^2^SCAN-3c level. The energies (given in kJ/mol) of the minima are relative to S1, and the energies of the transition states are relative to the minimum directly preceding it.

In total, the rotation about the single bonds that attach the benzoxazole moieties to the central C=C axle gives rise to four possible orientations of the benzoxazole rings with respect to each other in both the stable and the metastable conformations, leading to a total of eight ground-state minima. After subjecting the initial equilibrium distribution of stable conformations to light irradiation, the resulting photochemical *E-Z* isomerizations will give rise to a new distribution containing stable and metastable isomers. The distribution of metastable conformers will then thermally relax to reestablish the stable conformers’ thermodynamic equilibrium distribution. This process occurs through a combination of various possible pathways consisting of thermal helix inversions, single bond rotations connecting the central alkene and benzoxazole moieties, or a combination of both. An overview of all the possible pathways and their associated barriers is shown in Figure 3.

We performed low-temperature NMR experiments to support these computational findings (Figure S15). By cooling down a sample of **L** in THF-*d*_8_ to −100 °C, the signal in ^19^F NMR splits into four peaks corresponding to the four stable conformations, as a result of the impeded single-bond rotation involving the benzoxazole moieties on the NMR time scale. We have attempted *in situ* NMR irradiation under cryogenic conditions down to −100 °C to observe the formation of the metastable isomers. However, the THI barriers are too low to allow for sufficient buildup of this species to be detectable by NMR.

If alkene **L** behaves as a classical Feringa-type molecular motor, we would expect that a thermal helix inversion leaves the lower half unchanged in each of the metastable isomers, as depicted by the dashed light red arrows in Figure 3. However, to our surprise, such a transition state for this process could not be located. Instead, we found transition states in which this thermal helix inversion process is accompanied by either one or two single bond rotations involving the benzoxazole moieties, yielding stable conformers in which one or both benzoxazole moieties adopt an orientation that is different than the one in the metastable state from which it originates. The light red arrows indicate these processes. Additionally, both the stable and the metastable conformers can interconvert through single-bond rotations, which are indicated by blue arrows. There are two possibilities for each of these rotations, depending on whether the nitrogen atom or the oxygen atom in the benzoxazole rings passes the upper half. As expected, the electronic repulsion from the oxygen is larger than for nitrogen, generally giving higher barriers for rotation. Furthermore, in the stable conformers, the effect of the heteroatom is much larger in the transition states where the benzoxazole ring rotates past the naphthyl side of the upper half compared with the benzoxazole ring rotating past the quaternary center. In the metastable states, we could not identify such a trend.

The structures in the energy profile (Figure 3) were optimized at the r^2^SCAN-3c level of theory,^34,35^ considered one of the new standards for structural optimizations. The electronic energies of the optimized structures were also refined using MRSF TD-DFT^36^ (BHHLYP^37^ level with 6-31G*^38−40^ basis set), which can treat open-shell singlets without spin contamination. The results collected in Figure S2 follow the same trend as the ones computed with r^2^SCAN-3c, with notable differences only in the thermal *E*/*Z* isomerization. As shown in Figure 3, many transition states have similar energies, meaning that several pathways will take place at similar rates. For example, the photochemically generated metastable isomer **L-M1**, can undergo a direct THI, accompanied by two benzoxazole rotations, to the stable conformer **L-S1**. The barrier associated with this THI step is 47.2 kJ/mol. However, **L-M1** can also follow an alternative pathway in which it first undergoes a benzoxazole rotation to **L-M4**, followed by a THI with two benzoxazole rotations to **L-S2**, which finally undergoes another benzoxazole rotation to generate **L-S1**. The overall Gibbs free energy of activation for this process is 45.7 kJ/mol.

In our previous work, nanosecond transient absorption spectroscopy was performed on a sample of ligand **L** in CH_2_Cl_2_.^27^ Upon excitation of a sample of **L** with a 380 nm light pulse, a red-shifted transient is formed that relaxes back to the initial state with a lifetime of 1.5 μs. We attributed this signal to the thermal interconversion of the various conformers after the photochemical *E-Z* isomerization. With the current theoretical study, we can conclude that this observation matches the predicted behavior, and the lifetime of 1.5 μs is in excellent agreement with the calculated barriers for the various processes of benzoxazole-alkene single-bond rotations and THI steps, starting from the distribution of generated metastable conformers.

Besides the THIs and single-bond rotation, thermal *E*-Z isomerizations (TEZIs) should be considered (shown in pink in Figure 3). Broken-symmetry DFT calculations at the r^2^SCAN-3c level of theory were performed, after which the Yamaguchi formula was used to remove the spin contamination.^41,42^ These calculations show that the barriers for the TEZIs from the metastable conformers to their respective stable conformers are >70 kJ/mol. Therefore, these reactions are unlikely to significantly contribute to the thermal relaxation pathways.

### Excited State Dynamics

In Figure 3, the blue arrows represent photochemical *E*-*Z* isomerizations as expected for a typical Feringa-type molecular motor. However, upon observing the calculated structures of stable state **L-S1** and its corresponding metastable state **L-M1** (Figure 2), it is apparent that some degree of single bond rotation could also occur in the photochemical steps. Hence, one can imagine that during the photochemical *E*-*Z* isomerization, single-bond rotations–on the excited-state potential energy surface or in the vibrationally “hot” ground state–can also lead to various orientations of the benzoxazole rings. This hypothesis implies that each stable conformer can give rise to a mixture of metastable states upon irradiation with light.

**Figure 4:**
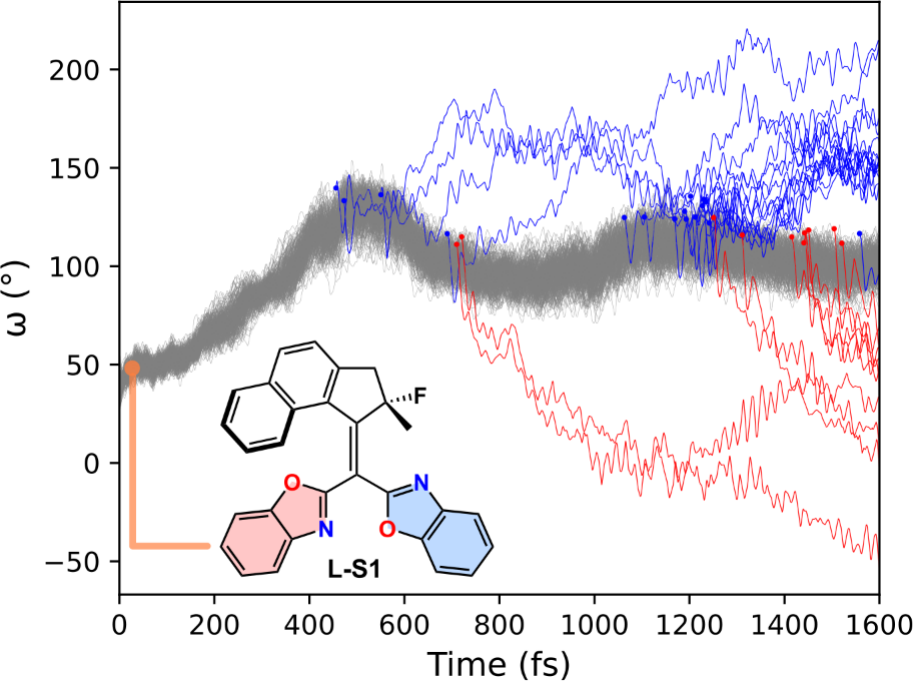
Changes in the dihedral angle between the upper and lower halves along 400 trajectories (**L-S1**) using excited-state molecular dynamics simulations at the OM2/MRCI level. A few trajectories that hop to the ground state are highlighted: ω_fin_ > 90° (blue line) and final ω_fin_ < 90° (red line). See Figure S4 for **L–S2-S4**.

We performed excited state calculations to elucidate the mechanism of the photochemical step, the distribution of metastable states it will give rise to, and the degree of unidirectionality in the photochemical part of the rotation cycle. Wigner sampling was performed on the optimized **L** stable conformer structures at the r^2^SCAN-3c level to obtain the different initial structures and relative initial velocities. The excited-state behavior was then modeled using molecular dynamics simulations at the OM2/MRCI(SD) level of theory^43,44^ which has been well benchmarked to investigate the excited state dynamics of molecular photoswitches such as azobenzenes and Feringa-type molecular motors.^45,46^ Furthermore, the method showed excellent agreement between theory and experiments in recent studies, demonstrating a good balance between computational cost and accuracy for excited state dynamics.^12,47^ Utilizing an NVT ensemble with a Nosé–Hoover thermostat at 300 K, following the initial excitation to the S_1_ state, 326 trajectories of **L-S1** were successfully propagated for 1.6 ps out of 400 initial structures. Immediately after excitation, the population moves away from the Franck–Condon point to a region where the double bond is fully broken, with ω of about 100° (see Figure 4) Although 26 trajectories successfully hopped to the ground state in the given time, the overall population remains in the perpendicular configuration even after 1.6 ps. Considering the long residence time of the molecule in this perpendicular region, we adopted a different approach to understand the evolution from the excited state to the ground state.

### Ground State Dynamics

From the analysis of the excited state dynamics, we anticipated that the population of the perpendicular state of the alkene was relatively long-lived (>10 ps). In this way, the molecules lose any coherent motion at the excited state and remain in a twisted configuration until they funnel to the ground state by reaching a conical intersection. We decided to follow an approach similar to the one published by Durbeej and coworkers,^48^ simulating the dynamics of the vibrationally “hot” ground state modeled by molecular dynamics simulations from the conical intersections (CInts).^48,49^ To approximate these geometries, the CInt structures (**L-CInt1**-**4**, Figure S1) were obtained at the GFN0-xTB^50−52^ level of theory using the 3.0 prerelease version of CREST,^53^ which uses a non-self-consistent xTB treatment. As expected, we optimized structures marked by a perpendicular configuration between the upper and lower halves (see Figure S1). These geometries were further validated using MRSF TD-DFT^36^ (BHHLYP^37^ level with 6-31G*^38−40^ basis set), resulting in excellent agreement with the ones obtained at the semiempirical level (Figure S5 and Table S1). To approximate the different geometries that the molecule can assume when reaching the conical intersection energy seam, we performed Wigner sampling for each CInt structure, yielding 400 distinct geometries per conformation of the conical intersection.^48^ We used these structures as the starting point of ground state molecular dynamics at the GFN2-xTB level^54^ (using Fermi smearing with an electron temperature of 1500K) for 10 ps with an integration time step of 0.25 fs, allowing us to investigate the conformational evolution following the internal conversion from S_1_/S_0_ CInt.

**Figure 5:**
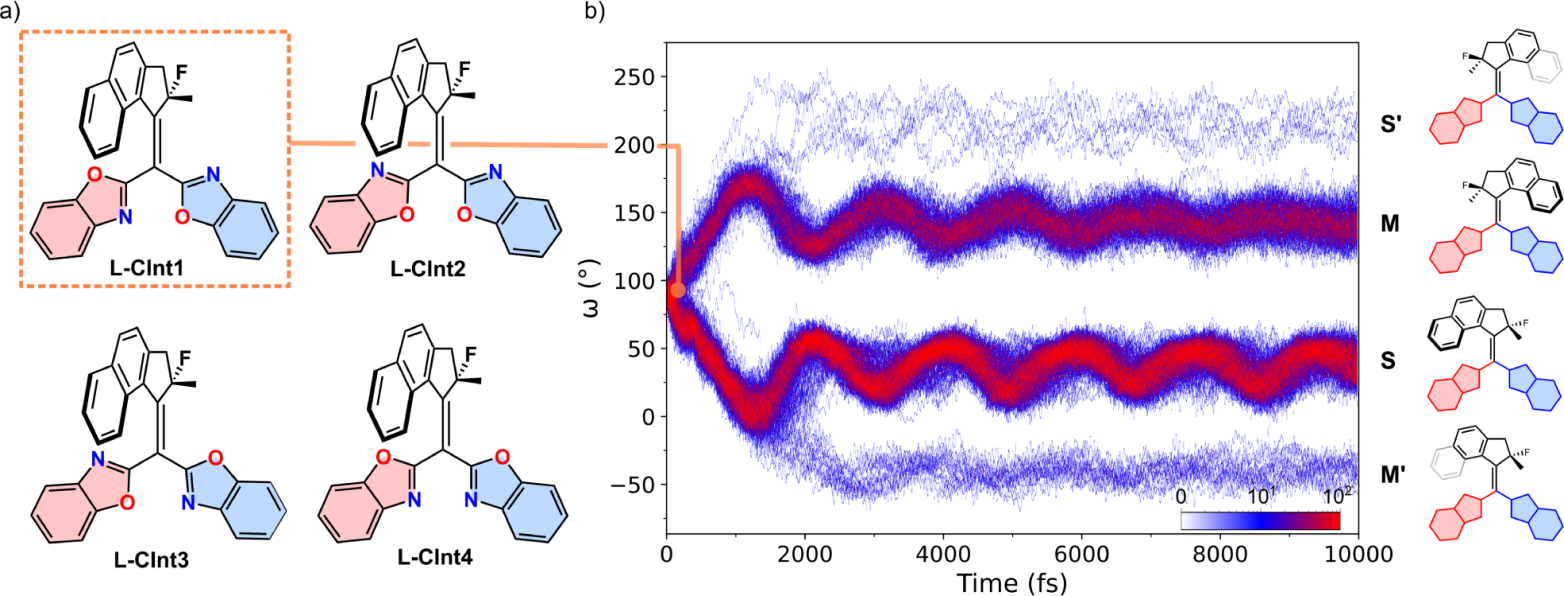
a) Configuration of **L-CInt1–4**. b) Changes in the dihedral angle between the upper and lower halves along 400 trajectories (**L-CInt1**) over 10 ps (see Figures S4–S6 for **L-CInt2–4**). The population was represented using a gradient color code from white to blue to red, corresponding to the increasing number of data points.

Throughout their evolution, we monitored the dihedral angle ω (associated with the C=C bond rotation) between the upper and lower halves. Initially, all dihedral angles were approximately 90°, while at longer time scales the population split into four distinct streams (see Figure 5). Based on the final value of the dihedral angle ω (ω_fin_), four distinct types of isomerization were identified and labeled as **M**, **S**, **M’**, and **S’** (Figures 5 and S6–S8). The trajectories with a final dihedral angle (ω_fin_) between 90° and 180° were classified as **M**, forming metastable state structures. In the context of conventional nomenclature in molecular motor studies, this could be considered as *productive isomerization* due to the formation of the metastable state following the photochemical *E*-*Z* isomerization. Conversely, trajectories with final dihedral angles (ω_fin_) between 0° and 90° were assigned to **S**, indicating the opposite direction of rotation compared to **M**. These trajectories produce stable state structures, representing *nonproductive isomerization* in the conventional context, reverting to the reactant that was excited in the photoisomerization process. **M’** (or **S’**) shows the same directional rotation as **M** (or **S**), but it is followed by a helix inversion allowing further rotation. In the case of **M’**, the molecule reverts to the original **S** state and has enough potential energy to continue its *backward* motion into the metastable conformer connected to the initial stable state by a THI step (see Figure 1). While this counterintuitive process seems to contrast with the concept of unidirectionality of the molecular motion, the relatively short time scales of the ground state isomerization do not allow to fully depict the complete scenario. Indeed, the thus formed metastable state will convert to the initial stable form via thermal helix inversion, overall adding to the population formed along the **S** trajectory. In a similar way, **S’** is composed by dynamics where the molecule has sufficient energy to not only undergo productive *E-Z* isomerization, but also invert its helicity via helix inversion. Mirroring the previous discussion, at longer time scales, this population will combine with the one obtained from the thermal helix inversion of **M** to form the next stable state after the initial reagent in a unidirectional fashion. In general, **M** and **S** are major isomerization paths, while **M’** and **S’** contribute to a minor extent (Table S2).

We found that the distribution of the final isomerization products is influenced by the initial orientation of the lower halves in **L-CInt1–4** (See Figure S1). When both nitrogen atoms of the benzoxazoles were aligned in the same direction (**L-CInt2** and **L-CInt4**), isomerization **M** was favored, with a proportion of 64–70%, (Table S2). In contrast, when the benzoxazole groups were oriented differently at the initial structures (**L-CInt1** and **L-CInt3**), isomerization **S** was more commonly observed, with a proportion of 54–60% (Table S2).

**Figure 6:**
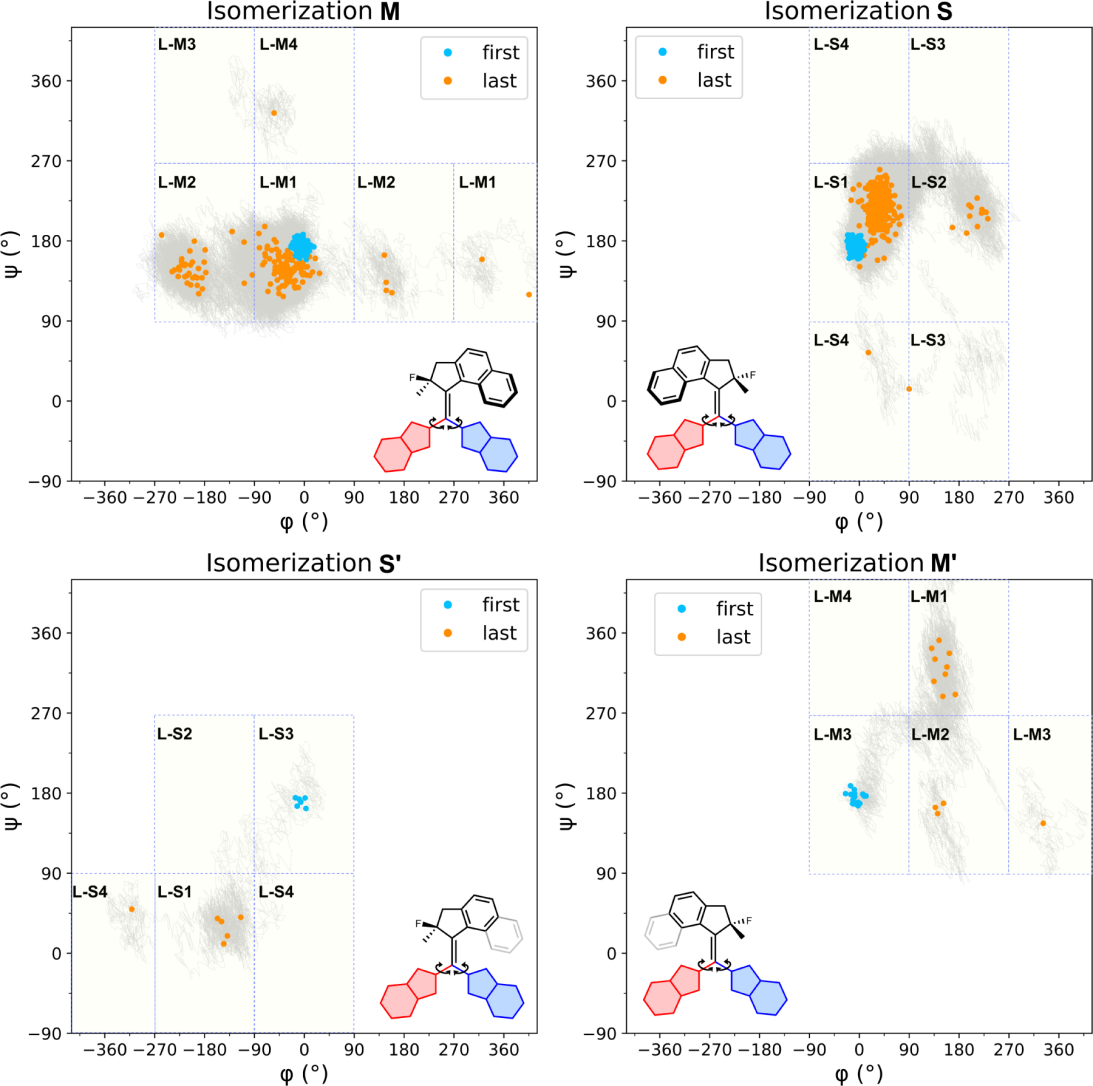
Changes in the dihedral angles φ, ψ from the initial angles (φ_in_ and ψ_in_) (blue dots) to final angles (φ_fin_ and ψ_fin_) (orange dots) along the pathways (gray line) during the dynamic process of **L-CInt1**. (See the Figures S9–S11 for **L-CInt2–4**). Each square represents a region corresponding to a specific conformer, meaning that a gray line or orange dot within a given square can be assigned to that particular conformer.

Notably, the C=C bond isomerization process can be accompanied by a single bond rotation in one or both lower halves. To better understand these motions, we plotted the trajectories of the dihedral angle changes in each lower half (indicated as φ and ψ) along the isomerization process (Figures 6 and S9–S11). For example, in the case of **L-CInt1**, the initial dihedral angles were φ_in_ ≈ 0° and ψ_in_ ≈ 180° (blue dots), the trajectories were tracked throughout the dynamic process (gray lines), indicating the final dihedrals as orange dots, allowing to assign the final structures to their corresponding conformers. We used grids (dashed-line boxes) to represent specific conformers in each area. Vertical shifts between sections indicate rotation of the right (blue) benzoxazole, while horizontal shifts correspond to rotation of the left (red) benzoxazole. A 180° rotation flips the benzoxazole upside down, resulting in a one-box shift. In the same way, a full-cycle rotation leads to a two-box shift. For example, during the isomerization **M** shown in Figure 6, several orange dots in two distinct **M2** areas were produced by the half-cycle rotation of the left benzoxazole through different directions. The two dots in the rightmost **M1** area were generated by a full-cycle rotation of the left benzoxazole, having the exact same orientation of the lower halves as the initial structure. Additionally, when the gray line spans or passes through a box, the corresponding conformer is formed transiently during the dynamic simulation.

**Figure 7:**
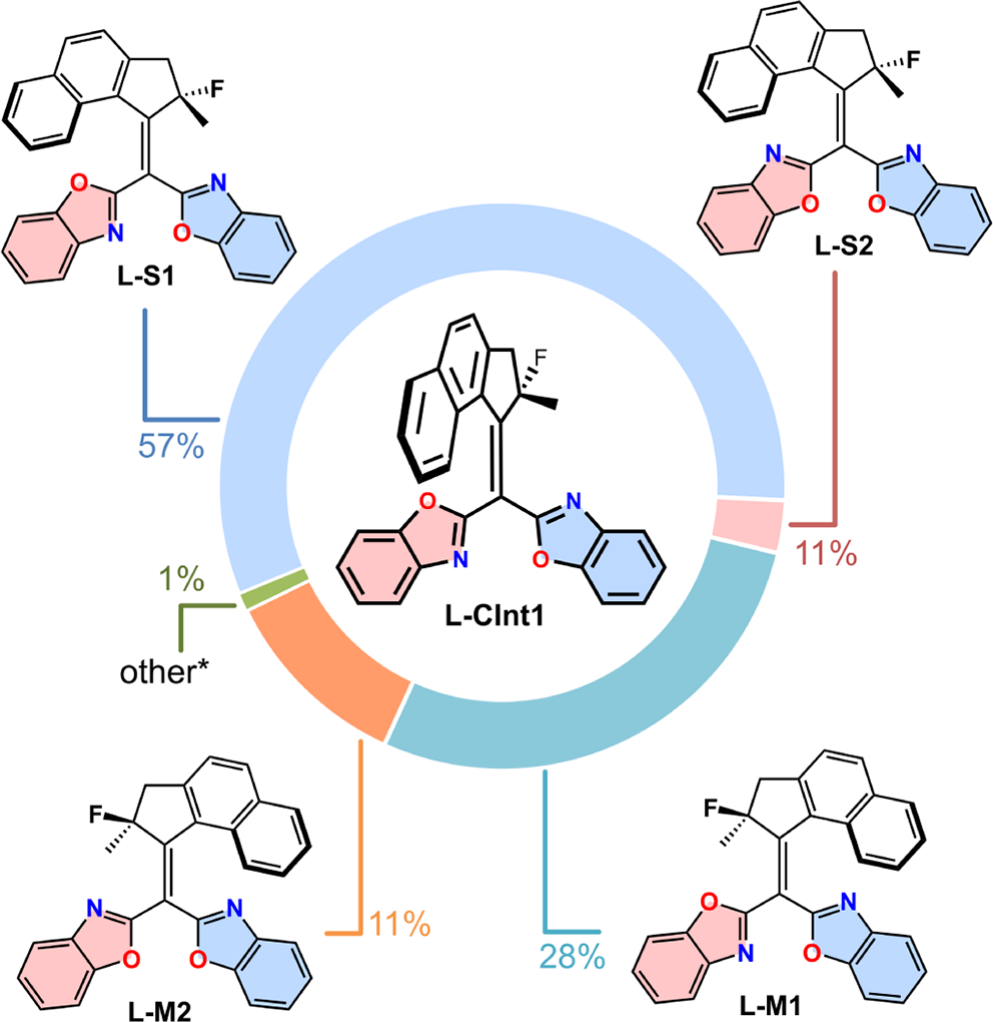
Distribution of conformers resulting from molecular dynamics on **L-CInt1**. *: **L-S3**, **S-S4**, **L-M3**, and **L-M4**.

Interestingly, a dependency of the single bond rotations on the type of isomerization was observed after summarizing the results from the ground-state dynamics (Figures 7 and S12–S14). For trajectories ending up in isomer **S**, it is shown that 89–94% of the cases have final structures in the same conformation as the initial structure, such as conversion from **L-CInt1** to **L-S1** (Table S2). In contrast, for trajectories that yield isomer **M**, merely 64–78% of the final structures also maintained the initial conformation. At the same time, a significant proportion of both the **S** and **M** trajectories ended up in the conformer of which the benzoxazole unit farthest from the naphthyl ring has rotated, when considering their final conformation. As an example, **L-CInt1** is converted to **L-M1** and, to a lesser extent, **L-M2**. Analogously, the major **M**-conformer yielded from **L-CInt2** is **L-M2**, with **L-M1** being the minor product. The other possible conformers constitute less than 1% of the final structures. The same relationship is true for **L-M3** and **L-M4**. We, therefore, conclude that the pairs of **L-M1** and **L-M2**, and of **L-M3** and **L-M4** show coupled motion, but the benzoxazole unit on the side of the naphthyl ring only rarely undergoes rotation during the vibrational relaxation from the conical intersection to the ground state.

An unsophisticated prediction of the quantum yields, using the ratios of **S** and **M** products (excluding the minor **S’** and **M’** products) for each conformer (Table S2), indicates that conical intersections **L-CInt2** (64%) and **L-CInt4** (61%) (and by extension **L-S2** and **L-S4**) are more efficient toward double-bond isomerization than **L-CInt1** (36%) and **L-CInt3** (46%). Hence, the *syn*-conformations, in which the heteroatoms in the benzoxazole rings adopt parallel orientations outperform the *anti*-conformations, in which the benzoxazole units adopt opposite orientations.

## Conclusion

In conclusion, our study provides a detailed theoretical understanding of the dynamic behavior of a bis(benzoxazole)-based overcrowded alkene, which exhibits both coupled and uncoupled molecular motions. Through a combination of excited- and ground-state molecular dynamics simulations, DFT calculations and NMR spectroscopy, we demonstrated that the photochemical *E*-*Z* isomerization of the central double bond of each stable conformer is directional. This process mirrors the mechanism of classical Feringa-type molecular motors, with the unique distinction that multiple pathways emerge during photochemical isomerization and the subsequent thermal helix inversion. We investigated the long-lived isomerization process (>10 ps) of **L** via molecular dynamics simulations combining Thiel’s Orthogonalization corrected methods and the more modern tight-binding approaches, covering the excited and ground state. In this way, we could better understand the directionality and interplay of molecular motions in a complex light-responsive structure such as **L**. Therefore, we anticipate that this approach and analysis will provide valuable insights and methodologies for helping the design and efficiency of enhanced, light-driven molecules. We envision that routinely affordable computations are a key step to understanding and eventually designing complex molecular movements in structures that can afford coupled motion.

## Supporting Information Available

Additional experimental details; computational methods; ^19^F NMR of compound **L**; interconversion pathways at the MRSF-TDDFT level; active space at the OM2/MRCI level; excited state trajectories for **L-S2–4**; comparison between conical intersections at the GFN0-xTB and MRSF-BHHLYP/6-31G^*^ level; ground state trajectories starting from **L-CInt2–4**; changes in dihedral angles for **L-CInt2–4**; final distribution of conformers for **L-CInt2–4** (PDF)
Optimized structures(ZIP)
