# Supplementary material for: Computational Study on the Dynamics of a Bis(benzoxazole)-Based Overcrowded Alkene
Source: J Phys Chem A. 2025 Jan 23;129(5):1301–9. doi: 10.1021/acs.jpca.4c06773 (PMC11808780; doi:10.1021/acs.jpca.4c06773)
Supplement: Supplementary file 1 — jp4c06773_si_001.pdf [file jp4c06773_si_001.pdf]

Computational Study on the Dynamics of a Bis(benzoxazole)-Based Overcrowded Alkene

Charlotte N. Stindt,[1‡] Taegeun Jo,[2‡] Jorn D. Steen,[2] Ben L. Feringa,[1*] Stefano Crespi[2*]

[1] Stratingh Institute for Chemistry, University of Groningen, Nijenborgh 4, 9747AG Groningen, The Netherlands
[2] Department of Chemistry - Ångström Laboratory, Uppsala University, Box 523, 751 20 Uppsala, Sweden

[‡]These authors contributed equally

Experimental methods

The synthesis of **L** as well as the ns transient absorption spectroscopy experiments and X-ray crystallographic data have been previously reported.[1] NMR spectra were recorded on a Varian Inova 500 (500 MHz) spectrometer.

Computational methods

The ground-state geometries were optimized using the ORCA 5.0.4 package[2] at the r^2SCAN-3c level of theory.[3] The nature of the stationary points found was confirmed computing the hessian matrix and inspecting the number of imaginary frequencies found (0 for the minima, 1 for the transition states). The nature of the thermal E-Z isomerization (TEZI) transition states was checked with a broken-symmetry calculation, after which the Yamaguchi formula was used to remove the spin contamination.[11,12] The TEZI transition state from **M2**-**S2** had a residual 2nd imaginary frequency of 9.83 cm^{-1}, which could not be removed. In order to evaluate the feasibility of the process, especially in light of the possible thermal E/Z isomerization, electronic energies were refined using MRSF (Mixed-Reference Spin-Flip) TD-DFT[4,5] in OpenQP[6] at the BHHLYP[7] level with the following basis sets: for H, Pople's 6-31G split valence basis set[8] ; for C and O, Pople's 6-31G split valence basis set[9] with polarization[10] (i.e. 6-31G*)
Nonadiabatic molecular dynamics simulations of the excited state were conducted at the OM2/MRCI level of theory,[13,14] as implemented in the MNDO program (MNDO2019, version 8.0). The active space in the MRCI calculations included ten electrons in ten orbitals (10,10). All the orbitals were of π character. For the MRCI treatment, three configuration state functions were chosen as references, namely the leading configuration with two singly occupied orbitals (which defines the ROHF formalism) and the two closed-shell configurations derived therefrom (i.e., the singlet configurations with doubly occupied HOMO or LUMO of the closed-shell ground state). The MRCI wavefunction was built by allowing all single and double excitations from these three references (CISD). Nonadiabatic molecular dynamics (NAMD) simulations on the different stable geometries were performed using the Tully surface-hopping (TSH) method as implemented in the MNDO program, with an analytical evaluation of the nonadiabatic coupling vectors.[15] All simulations were in a canonical ensemble maintained by a Nosé-Hoover thermostat (T=300 K). The sampling of the initial structures and relative initial velocities was obtained via a preliminary Wigner sampling on the geometry optimized at the r^2SCAN-3c level. The two lowest singlet states were included in the NAMD runs. The initial state chosen for starting the dynamics was S_1. 400 runs of 1600 fs with a time step of 0.1 fs were evaluated. The default empirical decoherence correction of 0.1 Hartree was used.[16]

The conical intersections were found at the GFN0-xTB level of theory using the 3.0 pre-release of CREST,[17] which utilizes a non-self-consistent xTB treatment.[18–20] These geometries were further validated using MRSF TD-DFT[6] (BHHLYP[7] level with 6-31G*[8–10] basis set), resulting in a good agreement (Figure S5 and Table S1). Following the method previously reported by Arpa, Stafström and Durbeej,[21] these obtained conical intersections were the starting points for the molecular dynamics simulations of the ground state at the GFN2-xTB level (using Fermi smearing with an electron temperature of 1500 K).[19,22] For each conformer (**L-CI-1–4**), 400 starting structures were generated by Wigner sampling from calculated harmonic vibrational frequencies. Based on these results, we compared the yields of conformers to investigate the directionality of **L** (Table S2). Unfortunately, tracking this process experimentally to validate the computational data is challenging because **L** exists as a mixture of conformers converting freely at room temperature (Figure S15): isolating a pure conformer from this mixture and subjecting it to selective irradiation is not feasible.

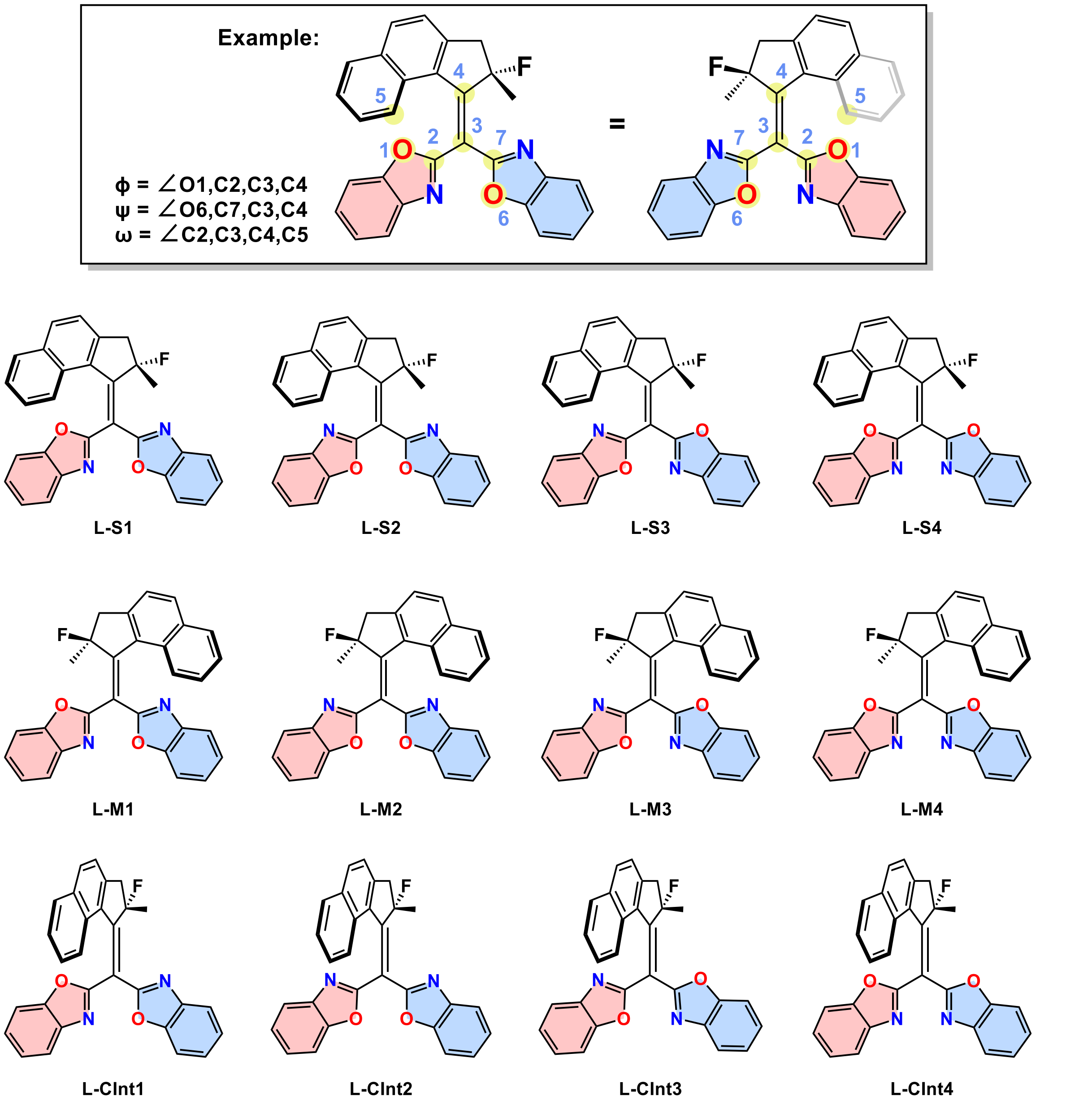

Figure S1. The conformers of the stable state (**L-S1-4**), metastable state (**L-M1-4**) and conical intersection (**CInt1-CInt4**), as well as the three characteristic dihedrals (ω, ϕ, ψ) used to assign the conformers.

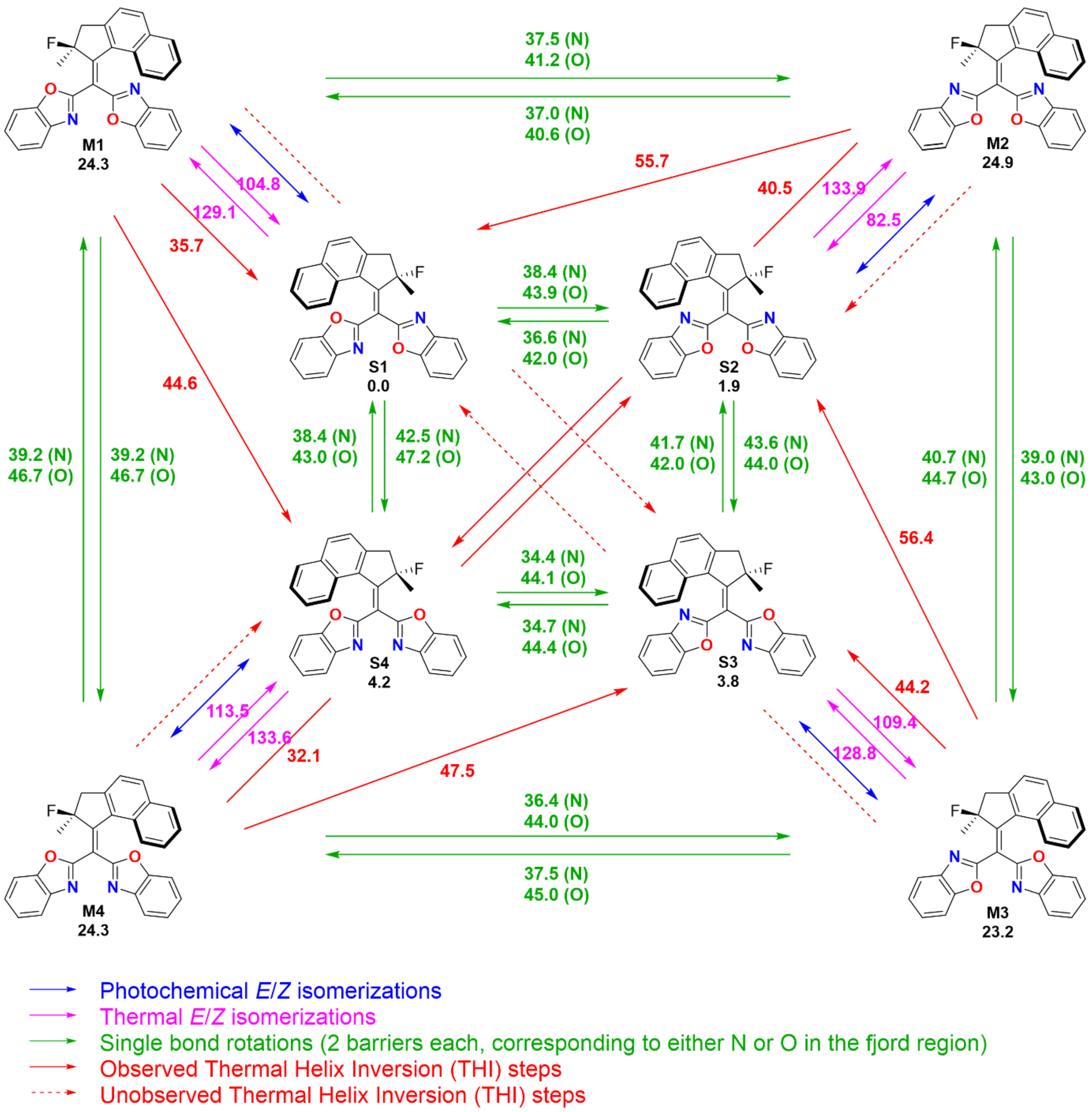

Figure S2. Overview of the possible interconversion pathways between the various stable (**S1** to **S4**) and metastable (**M1** to **M4**) conformation of compound **L**, through various *E-Z* isomerizations, single-bond rotations and THI steps. The structures were optimized at r^{2}SCAN-3c level and the energies (given in kJ/mol) were refined at the BHHLYP level with the 6-31G* basis set using MRSF (Mixed-Reference Spin-Flip) TD-DFT.

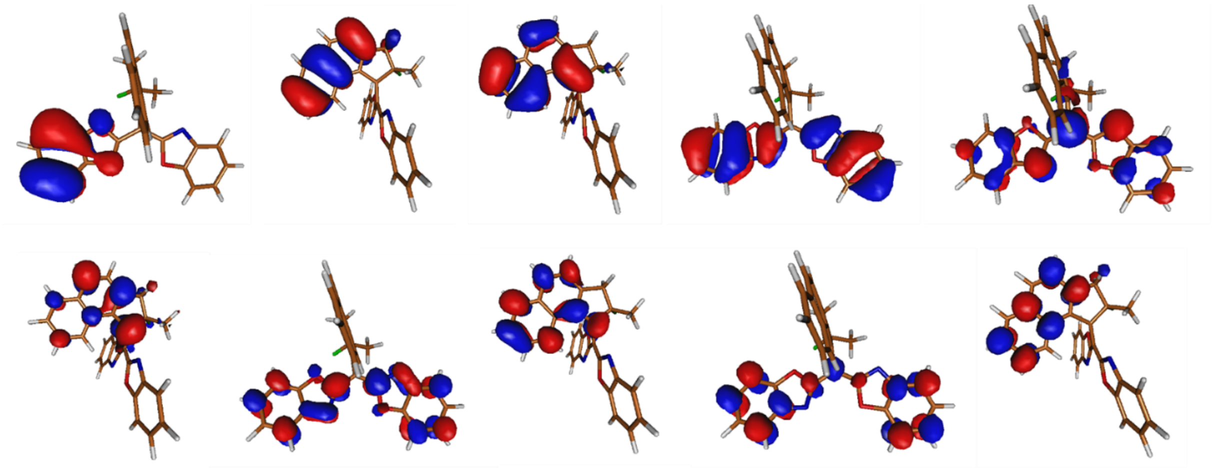

Figure S3. Active space orbitals employed in the OM2/MRCI calculations of **S1**.

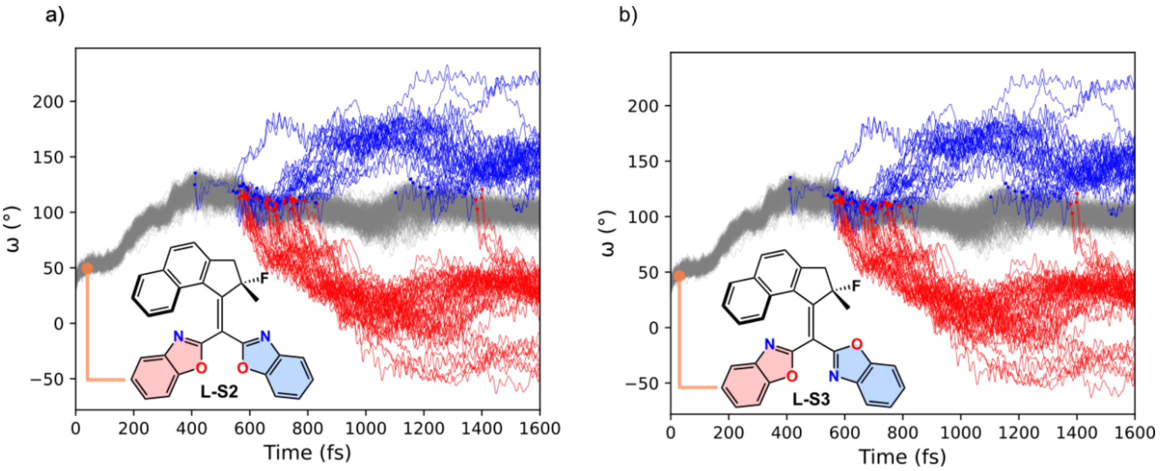

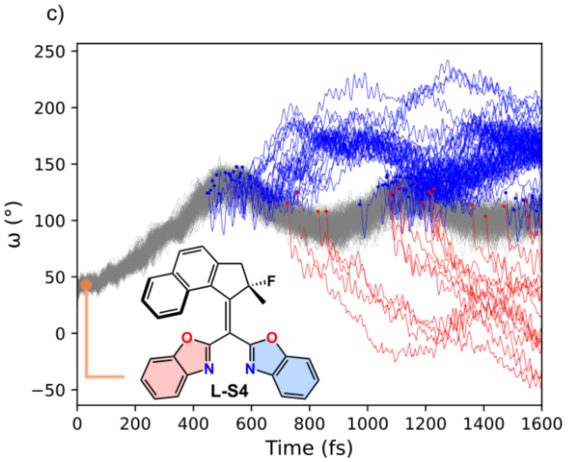

d)

	Hopping to the ground state	no hopping	Total cases
**L-S1**	26 (8%)	300 (92%)	326
**L-S2**	82 (22%)	287 (78%)	369
**L-S3**	156 (44%)	199 (56%)	355
**L-S4**	90 (25%)	263 (75%)	353

Figure S4. a), b) and c) Changes in the dihedral angle between the upper and lower halves along 400 trajectories (**L-S2-4**) using excited-state molecular dynamics simulations at the OM2/MRCI level. A few trajectories that hop to the ground state are highlighted: ω_{fin} > 90° (blue line) and final ω_{fin} < 90° (red line). d) The proportion between hopping and no hopping among the successfully propagated cases.

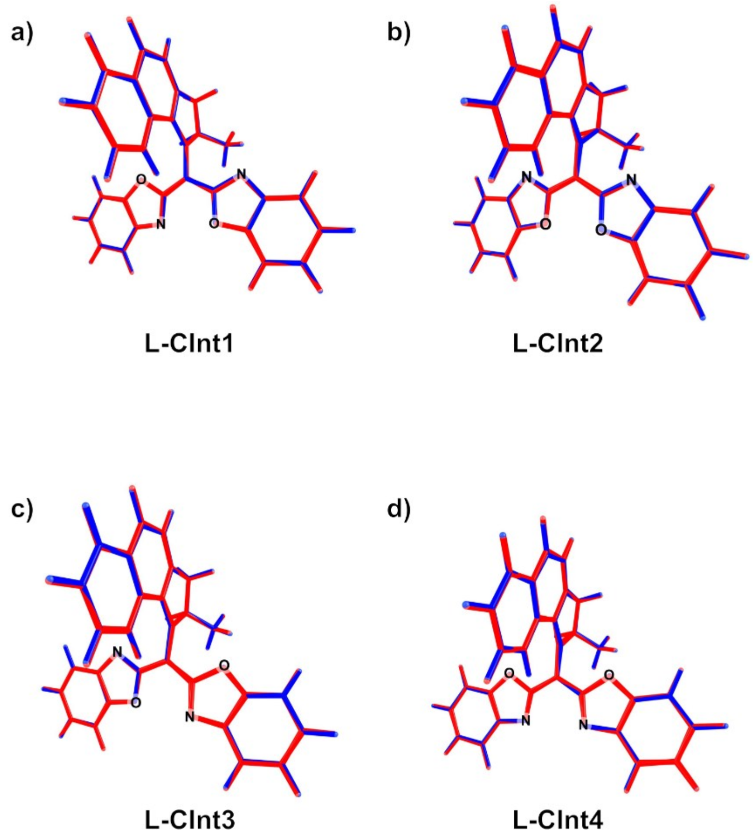

Figure S5. Overlay of **CInt1-4** optimized by GFN0-xTB (blue) and MRSF-BHHLYP/6-31G* (blue).

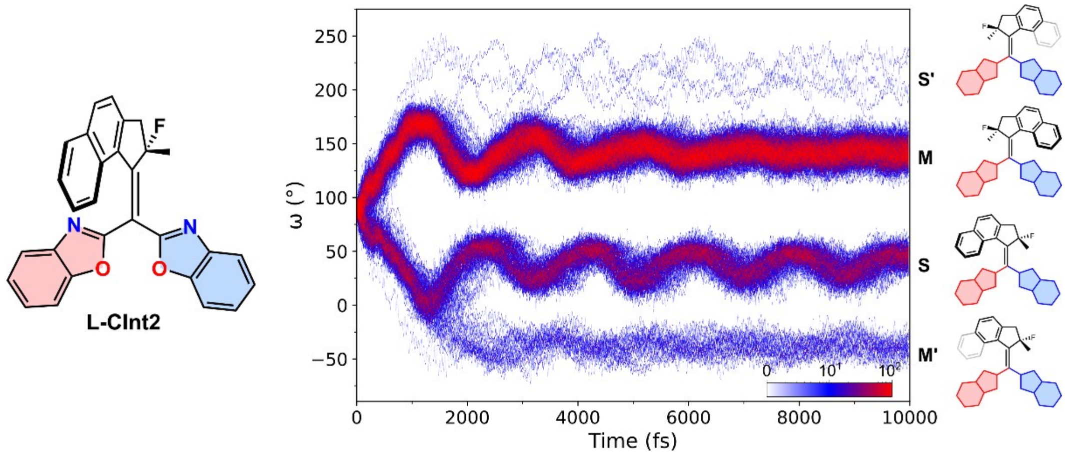

Figure S6. Changes in the dihedral angle between the upper and lower halves along 400 trajectories (**L-CInt2**) over 10 ps. The population was represented using a gradient color code from white to blue to red, corresponding to the increasing number of data points.

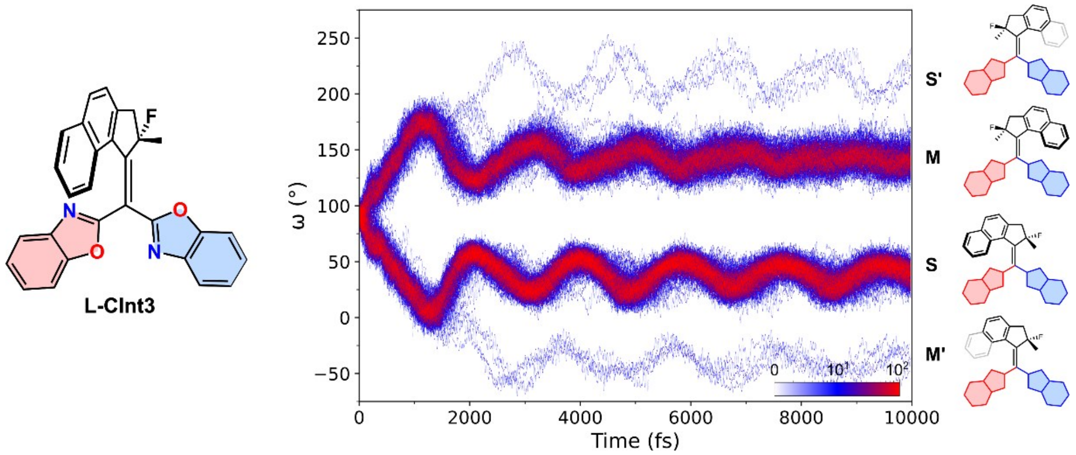

Figure S7. Changes in the dihedral angle between the upper and lower halves along 400 trajectories (**L-CInt3**) over 10 ps. The population was represented using a gradient color code from white to blue to red, corresponding to the increasing number of data points.

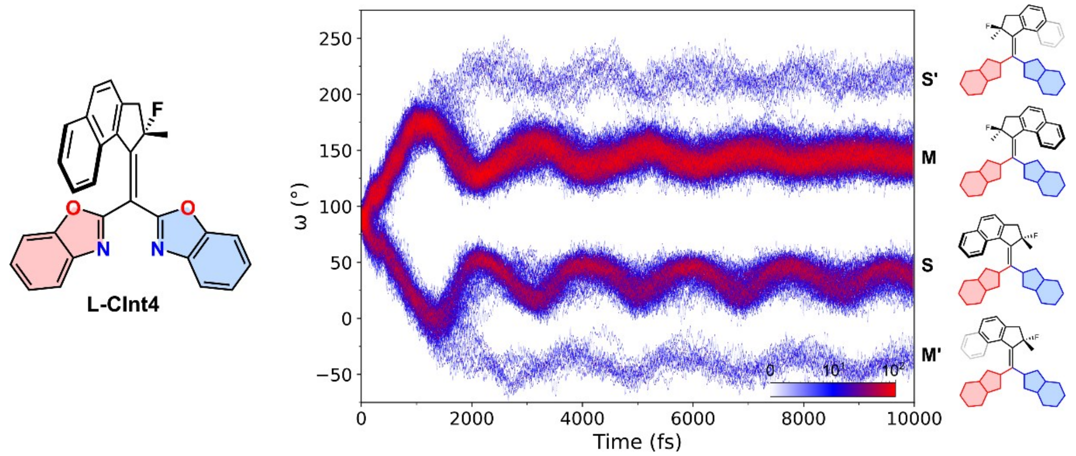

Figure S8. Changes in the dihedral angle between the upper and lower halves along 400 trajectories (**L-CInt4**) over 10 ps. The population was represented using a gradient color code from white to blue to red, corresponding to the increasing number of data points.

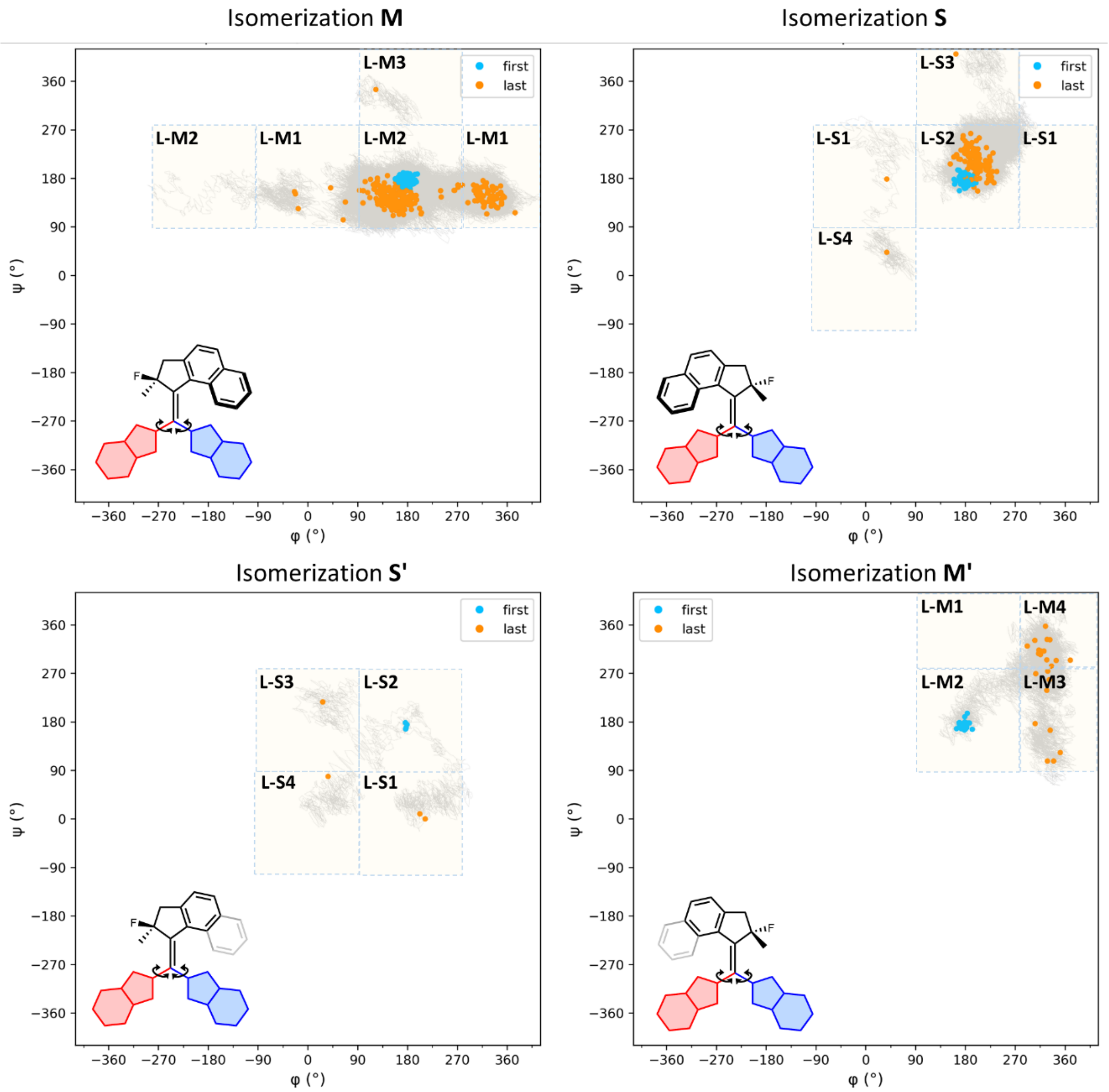

Figure S9. Changes in the dihedral angles ϕ, ψ from the initial angles (ϕ_{in} and ψ_{in}) (blue dots) to final angles (ϕ_{fin} and ψ_{fin}) (orange dots) along the pathways (grey line) during the dynamic process of **L-CInt2**. Each square represents a region corresponding to a specific conformer, meaning that a grey line or orange dot within a given square can be assigned to that particular conformer.

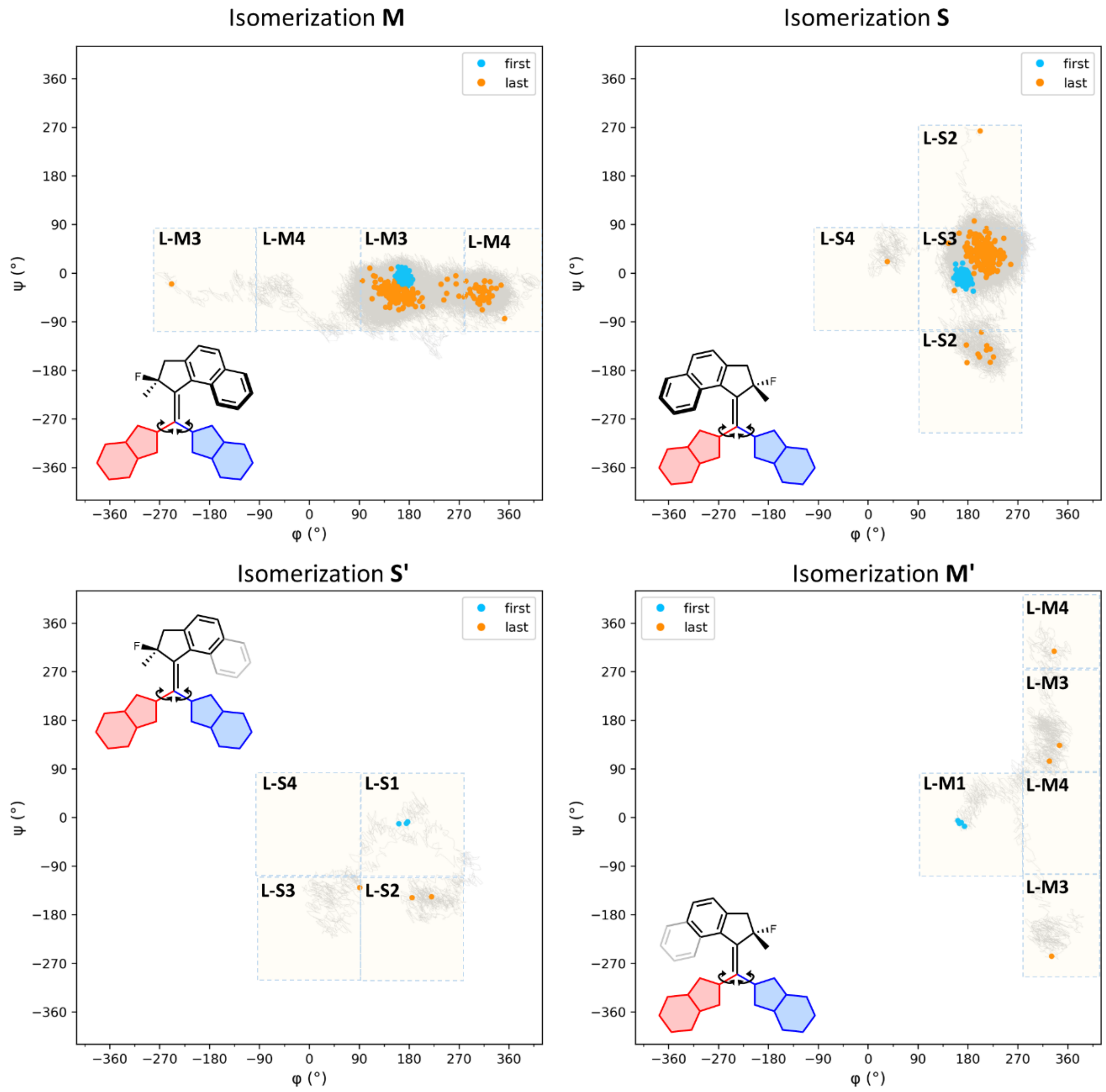

Figure S10. Changes in the dihedral angles ϕ, ψ from the initial angles (ϕ_{in} and ψ_{in}) (blue dots) to final angles (ϕ_{fin} and ψ_{fin}) (orange dots) along the pathways (grey line) during the dynamic process of **L-CInt3**. Each square represents a region corresponding to a specific conformer, meaning that a grey line or orange dot within a given square can be assigned to that particular conformer.

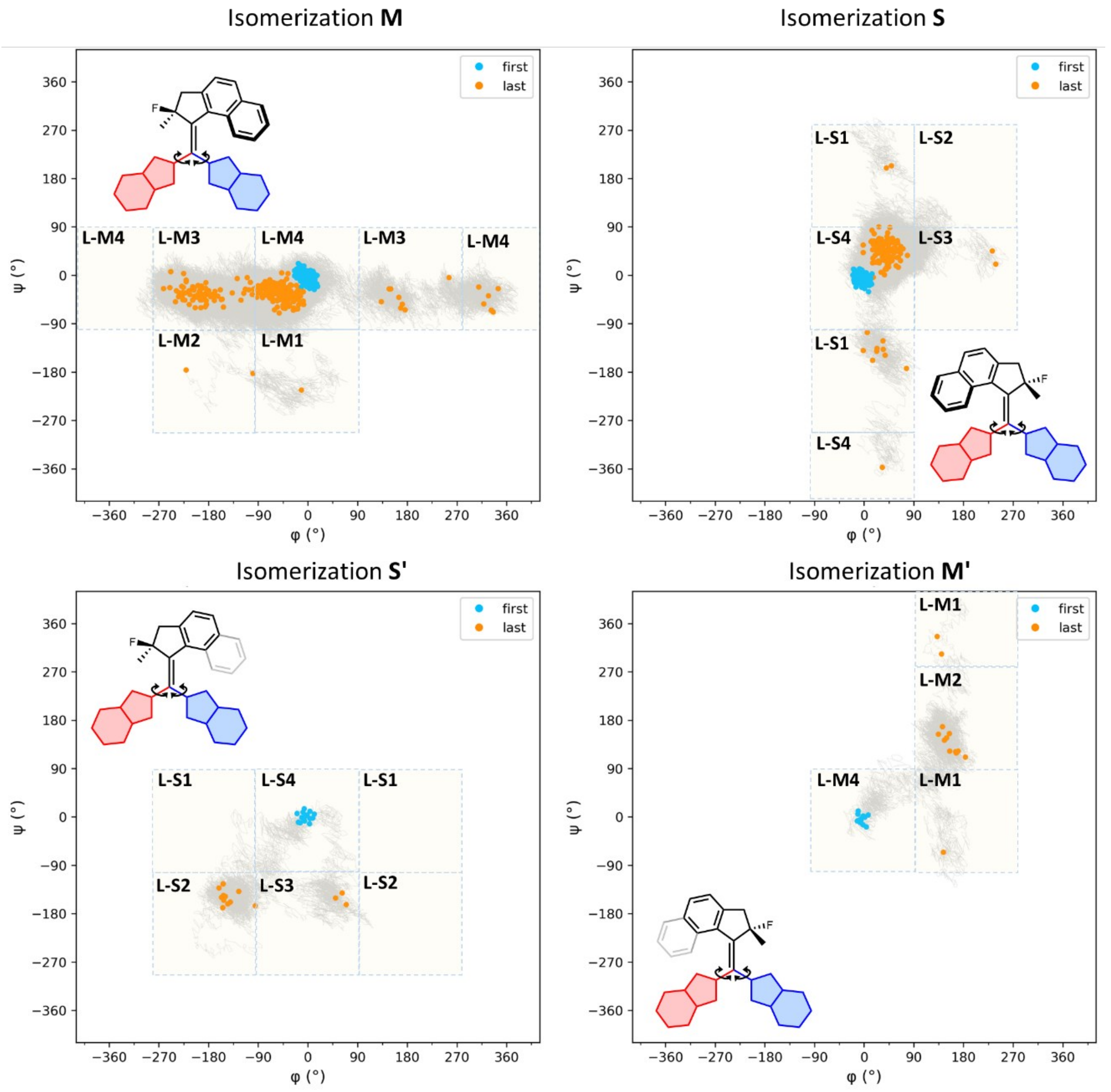

Figure S11. Changes in the dihedral angles ϕ, ψ from the initial angles (ϕ_{in} and ψ_{in}) (blue dots) to final angles (ϕ_{fin} and ψ_{fin}) (orange dots) along the pathways (grey line) during the dynamic process of **L-CInt4**. Each square represents a region corresponding to a specific conformer, meaning that a grey line or orange dot within a given square can be assigned to that particular conformer.

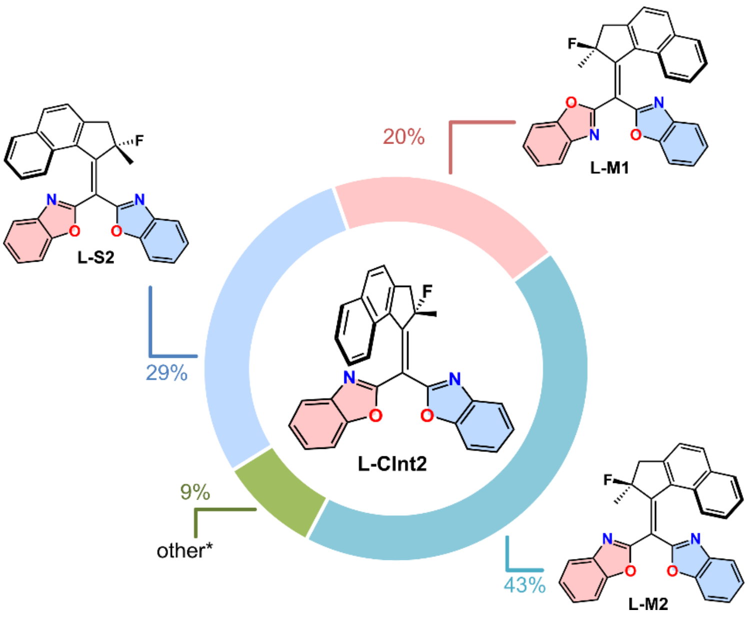

Figure S12. The distribution of conformers resulting from molecular dynamics on **L-CInt2**.

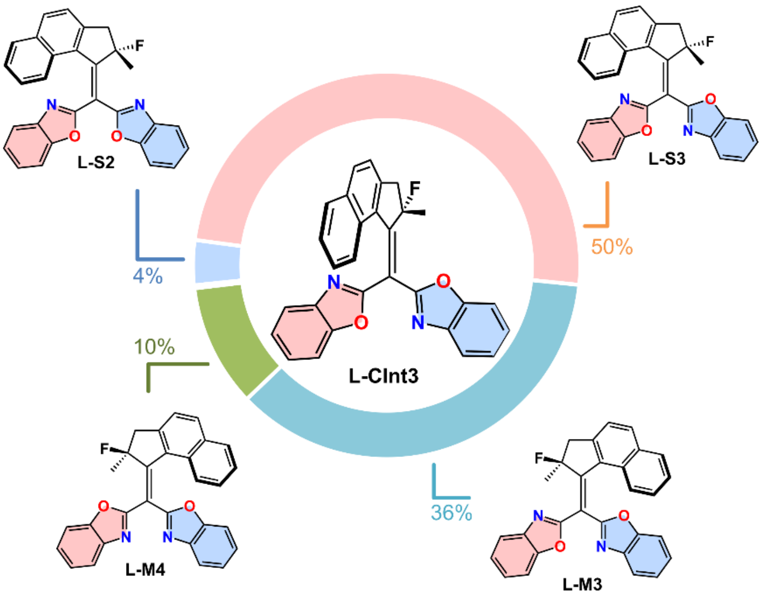

Figure S13. The distribution of conformers resulting from molecular dynamics on **L-Cint3**.

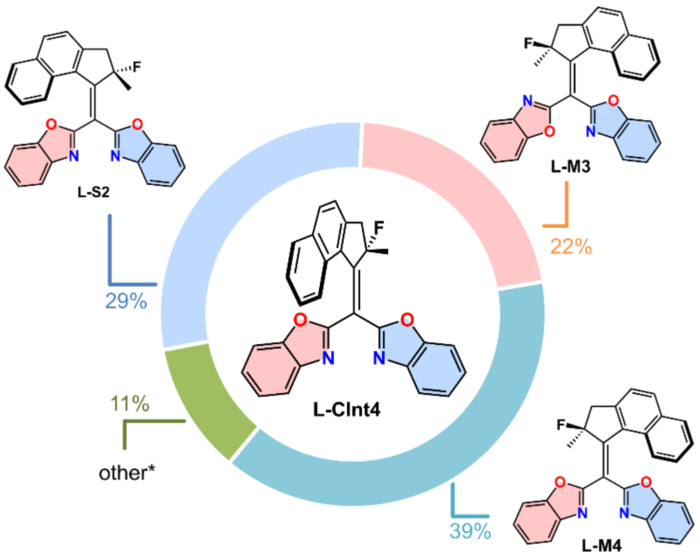

Figure S14. The distribution of conformers resulting from molecular dynamics on **L-CInt4**.

Table 1. Comparison of optimized geometries of **CInt1-4** by using the root mean square deviation (RMSD). Level of theories: GFN0-xTB (CREST) and BHHLYP level with 6-31G* basis set (MRSF TD-DFT in OpenQP).

Conical intersection	RMSD for GFN0-xTB *vs* MRSF-TDDFT (Å)
**CInt1**	0.047
**CInt2**	0.057
**CInt3**	0.049
**CInt4**	0.066

Table S2. The distribution of conformers resulting from molecular dynamics (**L-CInt1-4**)

**CInt1**			**S1**	**S2**	**S3**	**S4**	**M1**	**M2**	**M3**	**M4**
**M**	144	36%					104	39	0	1
**S**	234	59%	221	11	0	2				
**S’**	6	2%	5	0	0	1				
**M’**	16	4%					9	6	1	0
**Total**	**400**		**57%**	**3%**	**0%**	**1%**	**28%**	**11%**	**0%**	**0%**

**CInt2**			**S1**	**S2**	**S3**	**S4**	**M1**	**M2**	**M3**	**M4**
**M**	255	64%					81	173	1	0
**S**	118	30%	1	115	1	1				
**S’**	4	1%	2	0	1	1				
**M’**	23	6%					0	0	8	15
**Total**	**400**		**1%**	**29%**	**1%**	**1%**	**20%**	**43%**	**2%**	**4%**

CInt3			S1	S2	S3	S4	M1	M2	M3	M4
**M**	182	46%					0	0	142	40
**S**	211	53%	0	12	198	1				
**S'**	3	1%	0	3	0	0				
**M'**	4	1%					0	0	3	1
**Total**	**400**		**0%**	**4%**	**50%**	**0%**	**0%**	**0%**	**36%**	**10%**

CInt4			S1	S2	S3	S4	M1	M2	M3	M4
**M**	244	61%					1	2	86	155
**S**	129	32%	12	0	2	115				
**S'**	14	4%	0	11	3	0				
**M'**	13	3%					3	10	0	0
**Total**	**400**		**3%**	**3%**	**1%**	**29%**	**1%**	**3%**	**22%**	**39%**

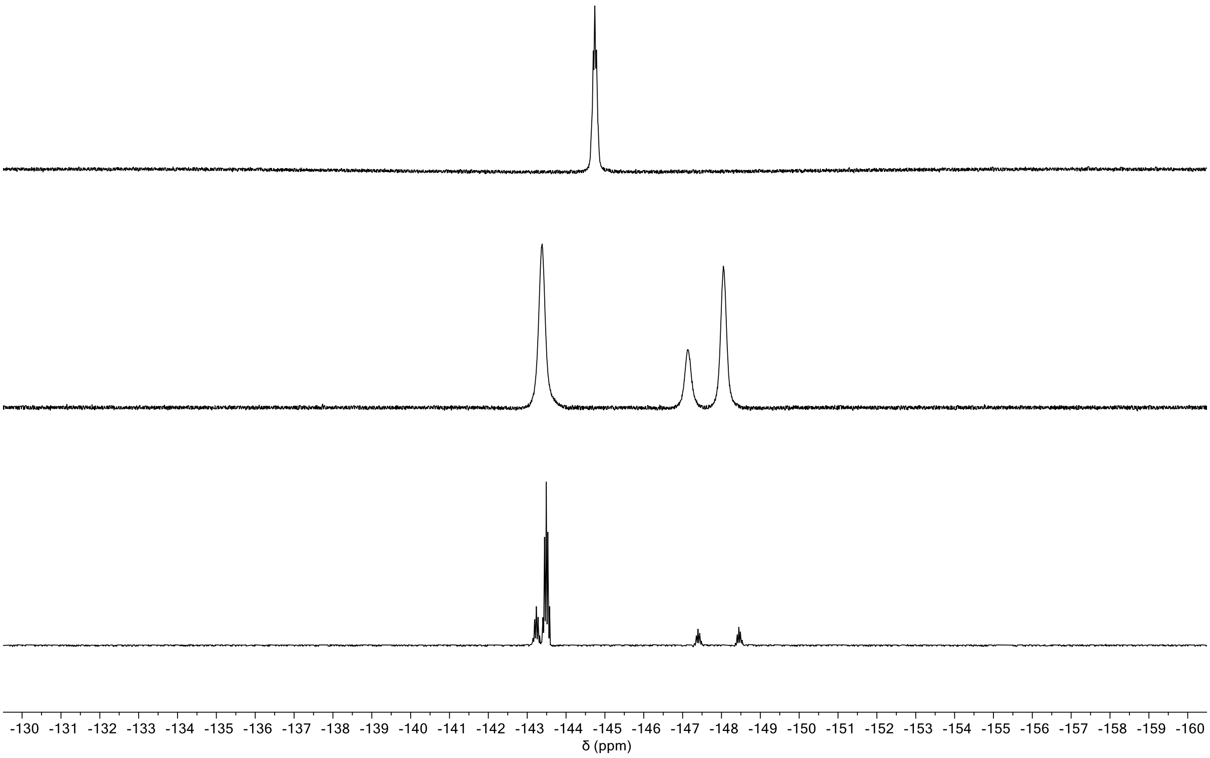

Figure S15. ^{19}F NMR (470 MHz) spectra of **L** in CD_2Cl_2 at RT (top), CD_2Cl_2 at −80 °C (middle) and THF-d_8 at −100 °C (bottom). The composition of the four conformers (downfield to upfield) is 19 : 66 : 7 : 8.

References

1 C. N. Stindt, S. Crespi, R. Toyoda, M. F. Hilbers, J. Kemmink, P. van der Meulen, W. J. Buma and B. L. Feringa, *Chem*, 2023, **9**, 2337–2348.

2 F. Neese, F. Wennmohs, U. Becker and C. Riplinger, *J. Chem. Phys.*, 2020, **152**, 224108.

3 S. Grimme, A. Hansen, S. Ehlert and J.-M. Mewes, *J. Chem. Phys.*, 2021, **154**, 064103.

4 S. Lee, M. Filatov, S. Lee and C. H. Choi, *J. Chem. Phys.*, 2018, **149**, 104101.

5 S. Lee, E. E. Kim, H. Nakata, S. Lee and C. H. Choi, *J. Chem. Phys.*, 2019, **150**, 184111.

6 V. Mironov, K. Komarov, J. Li, I. Gerasimov, H. Nakata, M. Mazaherifar, K. Ishimura, W. Park, A. Lashkaripour, M. Oh, M. Huix-Rotllant, S. Lee and C. H. Choi, *J. Chem. Theory Comput.*, 2024, **20**, 9464–9477.

7 A. D. Becke, *J. Chem. Phys.*, 1993, **98**, 1372–1377.

8 R. Ditchfield, W. J. Hehre and J. A. Pople, *J. Chem. Phys.*, 1971, **54**, 724–728.

9 W. J. Hehre, R. Ditchfield and J. A. Pople, *J. Chem. Phys.*, 1972, **56**, 2257–2261.

10 P. C. Hariharan and J. A. Pople, *Theoret. Chim. Acta*, 1973, **28**, 213–222.

11 K. Yamaguchi, F. Jensen, A. Dorigo and K. N. Houk, *Chem. Phys. Lett.*, 1988, **149**, 537–542.

12 N. Ferré, N. Guihéry and J.-P. Malrieu, *Phys. Chem. Chem. Phys.*, 2015, **17**, 14375–14382.

13 A. Kazaryan, Z. Lan, L. V. Schäfer, W. Thiel and M. Filatov, *J. Chem. Theory Comput.*, 2011, **7**, 2189–2199.

14 W. Thiel, *WIREs Computational Molecular Science*, 2014, **4**, 145–157.

15 E. Fabiano, T. W. Keal and W. Thiel, *Chem. Phys.*, 2008, **349**, 334–347.

16 G. Granucci, M. Persico and A. Zoccante, *J. Chem. Phys.*, 2010, **133**, 134111–134111.

17 GitHub - pprcht/crest at 3.0prerelease, https://github.com/pprcht/crest/tree/3.0prerelease, (accessed August 21, 2024).

18 P. Pracht, E. Caldeweyher, S. Ehlert and S. Grimme, *ChemRxiv*, 2019, preprint, DOI: 10.26434/chemrxiv.8326202.v1.

19 C. Bannwarth, E. Caldeweyher, S. Ehlert, A. Hansen, P. Pracht, J. Seibert, S. Spicher and S. Grimme, *WIREs Computational Molecular Science*, 2021, **11**, e1493.

20 P. Pracht and C. Bannwarth, *J. Phys. Chem. Lett.*, 2023, 4440–4448.

21 E. M. Arpa, S. Stafström and B. Durbeej, *Chem. Eur. J.*, 2024, **30**, e202303191.

22 C. Bannwarth, S. Ehlert and S. Grimme, *J. Chem. Theory Comput.*, 2019, **15**, 1652–1671.
